# A school-based health promotion programme to increase help-seeking for substance use and mental health problems: study protocol for a randomised controlled trial

**DOI:** 10.1186/s13063-016-1510-2

**Published:** 2016-08-08

**Authors:** Dan I. Lubman, Bonita J. Berridge, Fiona Blee, Anthony F. Jorm, Coralie J. Wilson, Nicholas B. Allen, Lisa McKay-Brown, Jenny Proimos, Ali Cheetham, Rory Wolfe

**Affiliations:** 1 Turning Point, Eastern Health, 54-62 Gertrude St, Fitzroy, VIC 3065 Australia; 2 Eastern Health Clinical School, Monash University, Box Hill, VIC Australia; 3 Melbourne School of Population and Global Health, The University of Melbourne, Parkville, VIC Australia; 4 Illawarra Health and Medical Research Institute, Wollongong, NSW Australia; 5 Graduate School of Medicine, University of Wollongong, Wollongong, NSW Australia; 6 Melbourne School of Psychological Sciences, The University of Melbourne, Parkville, VIC Australia; 7 Department of Psychology, University of Oregon, Eugene, OR USA; 8 Victorian Department of Education and Early Childhood Development, Travancore School, Travancore, VIC Australia; 9 Melbourne Graduate School of Education, The University of Melbourne, Parkvilleᅟ, VIC Australia; 10 Victorian Department of Education and Early Childhood Development, Melbourne, VIC Australia; 11 School of Public Health and Preventive Medicine, Monash University, Clayton, VIC Australia

**Keywords:** Prevention, Schools, Health education, Substance misuse, Alcohol, Young people, Wellbeing, Help-seeking

## Abstract

**Background:**

Adolescence is a high-risk time for the development of mental health and substance use problems. However, fewer than one in four 16–24 year-olds with a current disorder access health services, with those experiencing a substance use disorder being the least likely to seek professional help. Research indicates that young people are keeping their problems to themselves or alternatively, turning to peers or trusted adults in their lives for help. These help-seeking preferences highlight the need to build the mental health literacy of adolescents, to ensure that they know when and how to assist themselves and their peers to access support. The MAKINGtheLINK intervention aims to introduce these skills to adolescents within a classroom environment.

**Methods/design:**

This is a cluster randomised controlled trial (RCT) with schools as clusters and individual students as participants from 22 secondary schools in Victoria, Australia. Schools will be randomly assigned to either the MAKINGtheLINK intervention group or the waitlist control group. All students will complete a self-report questionnaire at baseline, immediately post intervention and 6 and 12 months post baseline. The primary outcome to be assessed is increased help-seeking behaviour (from both formal and informal sources) for alcohol and mental health issues, measured at 12 months post baseline.

**Discussion:**

The findings from this research will provide evidence on the effectiveness of the MAKINGtheLINK intervention for teaching school students how to overcome prominent barriers associated with seeking help, as well as how to effectively support their peers. If deemed effective, the MAKINGtheLINK programme will be the first evidence-informed resource that is able to address critical gaps in the knowledge and behaviour of adolescents in relation to help-seeking. It could, therefore, be a valuable resource that could be readily implemented by classroom teachers.

**Trial registration:**

Australia and New Zealand Clinical Trials Register (ANZCTR): ACTRN12613000235707. Registered on 27 February 2013.

**Electronic supplementary material:**

The online version of this article (doi:10.1186/s13063-016-1510-2) contains supplementary material, which is available to authorized users.

## Background

### Mental health and substance use are major health issues for young people

Adolescence is a high-risk time for the development of mental health and substance use problems. Indeed, half of all lifelong mental disorders (including substance use disorders) commence by the age of 14 years, with three quarters beginning before the age of 25 [1]. In Australia, over a quarter of 16–24 year-olds meet criteria for a mental disorder in the previous 12 months, with anxiety (15.4 %), depression (6.3 %) and substance use disorders (12.7 %) being the most commonly experienced conditions [2]. Despite cross-sectional data showing an increase in non-drinking among Australian adolescents [3], harmful use of alcohol is still the most common and concerning substance use issue among this cohort. The most recent Australian Secondary Students Alcohol and Drug Survey identified that just over half of all 12–17 year-olds had consumed alcohol in the past year, and 50.7 % of this group were drinking at harmful levels by age 17 [4]. Equally concerning, in 2013, surveys showed that around one in six people aged between 12 to 18 had consumed 11 or more standard drinks on a single drinking occasion in the past 12 months [5]. Untreated mental health issues and early onset substance use often co-occur and can lead to a range of short-term harms. Ultimately, substance use can adversely impact relationships, educational and developmental milestones, as well as later mental and physical health [6].

### Young people are reluctant to seek professional help

Seeking help early is widely recognised as a generic protective factor, and promoting early and prompt treatment is critical in order to reduce the adverse impacts of mental health and substance use problems [6]. However, fewer than one in four 16–24 year-olds with a current disorder access health services, with those experiencing a substance use disorder being the least likely to seek professional help [2]. Rather than seeking professional help, research indicates that young people are keeping their problems to themselves or turning to their peers or key adults in their lives for help [7]. This is despite evidence that many parents and peers have poor mental health literacy [8], as indicated by their limited ability to recognise specific disorders, poor knowledge of how to seek mental health information, and poor knowledge of risk factors and causes, self-treatments and professional help available, as well as attitudes that do not promote recognition and appropriate help-seeking [9]. In addition, adolescents have knowledge, attitudes and beliefs about help-seeking and substance use that act as barriers to seeking professional help, and these are likely to have been established before the age of 13 [10]. Barriers identified include stigma, fears about lack of confidentiality, limited trust, lack of problem recognition, reliance on oneself, and concerns about helper characteristics. These help-seeking beliefs and preferences highlight the importance of building the mental health literacy of adolescents, including ensuring that they know when and how to assist their peers to access support.

### Schools are ideal sites for health promotion activities and strengthening gatekeeping skills

Schools are an ideal and opportunistic setting in which to reach out to young people [11], particularly in terms of facilitating future help-seeking for mental health and substance use issues. Given the low help-seeking intentions of adolescents, teachers and peers are ideally placed to play a gatekeeping role (i.e. identifying issues and intervening), by supporting and helping young people to access appropriate professional support. To be effective, gatekeepers require the skills to identify mental health issues, engage the young person and help them overcome the barriers to accessing and engaging with professional help. Additionally, there is evidence from the mental health first-aid literature that teaching people how to help their peers seek help not only improves gatekeeping skills but is an innovative approach to improving their own mental health and help-seeking attitudes [12].

### Current gaps in the curriculum and opportunities to intervene

Although some school drug and mental health education programmes have been produced that focus on youth participation and peers as educators [13, 14], to our knowledge these have generally not focussed on exploring the barriers to helping a friend, taught students the skills necessary to overcome these barriers, nor facilitated professional help-seeking. In short, they do not focus on teaching practical steps for peers to become effective gatekeepers for their friends, which in turn, would signify that they have become proficient in terms of their own help-seeking skills. To this end, we previously reported findings from a pilot help-seeking intervention study (MAKINGtheLINK), with a focus on cannabis use and mental health, delivered over two 48-minute periods to 10 year-10 classes (182 students) at a Melbourne high school, as part of their standard curriculum [15]. The delivery of the programme was found to be both acceptable and feasible within a school setting, with students reporting increased confidence and awareness of how to seek help for themselves or a friend. A second pilot, which focussed primarily on help-seeking for alcohol and mental health, was conducted over three 50-minute periods, to 16 year-8 and year-9 classes (370 students) from three Melbourne high schools, as part of their standard curriculum [16]. Students reported that the programme led to increased knowledge about alcohol, awareness of help-seeking options and confidence to seek help for an alcohol problem. While these findings are encouraging, the pilot studies included few schools, without a control arm, and did not measure any change in actual help-seeking. Before the programme can be finalised and embedded within a national school framework, the efficacy of the programme needs to be established utilising a rigorous methodological and longitudinal design.

### Aims and hypotheses

This cluster randomised trial seeks to demonstrate the efficacy of a universal, school-based intervention that focusses on reducing barriers and improving help-seeking and peer support for students who are experiencing poor mental health and/or misusing alcohol or other drugs.

The primary hypothesis to be tested is that:

- Participation in the intervention, compared to a waitlist control group, will lead to increased help-seeking behaviour (from both formal and informal sources) for alcohol and mental health issues at 12 months post intervention, as measured by the Actual Help Seeking Questionnaire

We will also test the following secondary hypotheses:

- Compared to a waitlist control group, participation in the intervention will lead to increased confidence to seek help immediately after, and 6 and 12 months post intervention as measured by the General Help Seeking Questionnaire
- Compared to the control group, participation in the intervention will lead to increased confidence to assist a peer to seek help immediately after, and 6 and 12 months post intervention as measured by the General Help Seeking Questionnaire and self-reported help-seeking
- Compared to the control group, participation in the intervention will lead to a reduction in psychological barriers for help-seeking associated with alcohol and depression immediately after, and 6 and 12 months post baseline, as measured by the Barriers to Adolescents Seeking Help – Brief Version questionnaire

## Methods/design

### Study design

**Fig. 1:**
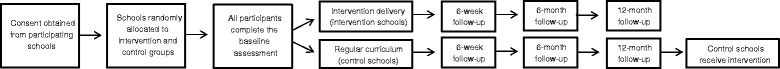
Schematic illustration of the research design

The study (see Fig. 1) is a randomised controlled trial (RCT) with schools as clusters and individual students as participants, and will follow the Standard Protocol Items: Recommendations for Interventional Trials (SPIRIT) Statement for reporting trial protocols (see Additional file 1 for checklist) [17]. All consenting participants will be assessed at four time points: baseline, 6 weeks post baseline, 6 months post baseline and 12 months post baseline. Participants from the control schools will be assessed on equivalent dates at the four time points.

Twenty-two Victorian secondary schools will be recruited for the study. Schools will be randomly allocated to either receive the intervention immediately or to form a waitlist control group and receive the intervention after completion of the fourth survey. Randomisation will be stratified by the school’s Index of Community Socio-Educational Advantage (ICSEA) score, with two strata defined as <1000 (‘disadvantaged’) and 1000+ (‘advantaged’). The random allocation list will be generated using Stata statistical software, Release 12 (StataCorp, College Station, TX, USA: 2011) with random block sizes of 2 or 4 within each strata.

### Sample

Eligible participants are year-9 students (aged 14–15 years) who consent to participate in the study. Eligible schools are state, Catholic or independent schools in Victoria with between 40 and 250 year-9 students. Schools will be sent an email explaining the study and inviting participation. To be included in the sample, schools will have to be willing to support their 2013, 2014 or 2015 year-9 cohorts to participate in the health education programme offered as part of this research, as they will not know in which year they will receive the programme until randomisation and consent form collection has occurred.

### Sample size calculation

Sample size calculations accounting for cluster randomisation have been estimated using Stata 12 software (StataCorp, College Station, TX, USA: 2011). Useful background information is available from a controlled pre-post pilot study conducted in three regional high schools that were matched on socioeconomic characteristics [18]. The trial group included 171 year-11 students from mainstream classes who received a school-based intervention (Building Bridges to General Practice) that addressed adolescents’ psychological help-seeking barriers in presentations delivered by trained general practitioners (GPs) [18]. At baseline, only 1 (0.6 %) of the 171 students reported visiting a GP. At 12-week follow-up, 12 students (7.0 %) indicated receiving a consultation. Assuming the pre-test consultation rate represents the control group post measure for the proposed trial, we estimated the effect size in schools (where the intervention is implemented by trained staff as the expected difference in proportions between the control arm and the intervention arm) to be 6.4 % (pooled SD 18.8 %). Assuming 80 % successful consent and randomisation, followed by a 25 % attrition rate between baseline and follow-up, the number of students in the analysis would be 60 % of a school’s year-9 level. We anticipate that this will lead to an approximate average cluster size for analysis of 51 students per school.

It is important to note that the assumed effect size may be a conservative estimate considering the intervention’s focus on help-seeking from both formal and informal sources, rather than just GPs, who young people are reluctant to access. In other words, both the prevalence at baseline and the difference in proportions at 12-month follow-up, of students seeking help from formal and informal sources are likely to be greater than the pilot study indicates for seeking help from GPs.

For the proposed project, the intra-class correlation (ICC) is unknown, although a comparable school-based cluster randomised trial conducted by Jorm and colleagues reported an ICC of 0.05 [19]. Setting ICC (ρ) at 0.05, power (1 − β) at 80 %, and a two-sided level of significance at 5 % (*α* = 0.05), the required number of individuals per treatment arm is 476 students corresponding to a need for 10 schools per arm. The desired sample size was 1020 and this was increased to 1360 to allow for 25 % dropout by the 12-month follow-up assessment.

### Intervention

The help-seeking intervention draws upon two models of behaviour change – the Information-Motivation-Behavioural Skills Model (IMB) and the Theory of Planned Behaviour (TpB). The IMB model is a well-validated, comprehensive health behaviour change framework that has been used in schools, particularly for HIV education and prevention [20]. The TpB is a health behaviour change framework that has been used extensively to guide experimental health intervention trials [21]. In this study, the help-seeking activities to be trialled provide students with information about how to seek help and from whom (Information), investigate participants’ psychological barriers to help-seeking (Beliefs, Intentions), investigate risky behaviours associated with alcohol use and mental health problems (Symptom levels), and provide opportunities and videos for skill rehearsal (Behavioural skills) which, according to our composite model, will lead to increased intention to seek help and actual help-seeking (Behavioural outcome).

The intervention will consist of five interactive classroom activities run over two school periods (average period is 75 minutes), plus a booster session 1 month later (to reiterate key messages and help students gain practical experience by applying the help-seeking skills they have learnt), delivered by an experienced external facilitator with the assistance of the regular classroom teacher. The rationale for the addition of a booster session is based on feedback and data from our pilot investigations, as well as research indicating the importance of booster sessions in terms of enhancing and maintaining treatment effects [22]. Activities will cover (1) recognising when a friend needs help (vignettes about poor mental health and risky drinking), (2) what types of helpers are available, (3) myths and facts about substance use and mental health, (4) identifying and overcoming barriers to professional help-seeking, (5) assisting a friend to access help, and (6) accessing reliable sources of help (see Table 1).

**Table 1:** Summary of activities included in the intervention

Activity	Description
Ranking for risk	*Know* mental health can fluctuate from ‘good’ to ‘poor’ in the course of a day or over a lifetime the difference between poor mental health and mental illness relates to duration and severity of symptoms *Understand* people perceive risk differently depending on their experiences, values and beliefs a drug experience will differ depending on the drug that is taken, the person who takes it and where they take it talk about suicide must be taken seriously and the friend at risk must be referred on to an adult *Do* a student is able to assess short-term and long-term risk in a hypothetical situation identify ‘red flags’ (e.g. risk of harm to self or to others, change in behaviour, etc.)
Myths and facts	*Know* what the research states in relation to the selected ‘myths’ and ‘facts *Understand* that stigma and misunderstandings relating to mental health issues and substance use do exist and these may act as barriers for young people thinking about seeking help *Do* correct misperceptions held by peers around mental health and substance use
Under construction	*Know* the brain is still developing until the age of 25 years *Understand* what happens to the adolescent brain and the body when alcohol is consumed *Do* a student is able to make informed choices about drinking alcohol as an adolescent
Helpers	*Know* the range of helpers that are available within and outside of school *Understand* the barriers that may stop a person from seeking help the difference between professional confidentiality and duty of care *Do* a student is able to ask questions to confirm what information will be passed on, and what will be kept confidential when speaking to a helper
Helping Jason	*Know* how to have an effective health-seeking conversation with a friend *Understand* that encouraging a friend to seek help may take time, and repeated efforts that it is more effective to ask questions than to tell someone what to do *Do* a student is able to plan a help-seeking conversation with a friend
Booster	*Know* key information from the course (see previous activities) *Understand* key information from the course (see previous activities) *Do* ‘sell’ a health-promoting message to a friend (in poster form) tasks as specified in the above five ‘do’ categories

### Recruitment process

Recruitment for the trial began in August 2013. Emails providing information about the programme opportunity and inviting expressions of interest from schools were sent to all Catholic, independent, and state schools within 50 km of Melbourne’s CBD with a year-9 cohort of between 40 and 250 students. Schools were required to obtain consent forms signed by the students’ parent/guardian for them to participate in the study, returning a minimum of 60 % positive consents of year-9 students and guardians to be eligible to participate in the trial. In order to maximise return of consent forms, each school principal assigned a staff member to the MAKINGtheLINK project in order to take responsibility for their collection. In addition, the research team ran two presentations for year-9 students to explain why participation in the survey was important, provided clear instructions about where and when to return consent forms, and provided extra copies of forms where required. After these initial steps were taken, contacts at each school emailed or telephoned parents to follow up with them about the consent forms.

In spite of these efforts, we were unable to obtain the required sample size at four of the seven schools that had agreed to participate by December 2013. In order to increase participation rates, and supported by the Department of Education and Early Childhood Development, a passive/opt-out consent process was adopted from 2014 onwards where parents were no longer required to opt in to the research, and students could make an informed choice as to whether they wanted to participate. Under this process, students are assured that participation is optional and that they will not be penalised for choosing not to participate, and are required to check the ‘consent’ box before completing the online survey. Parents are informed of the research at least 1 month in advance and can opt out of the research trial by contacting the school if they do not want their children involved.

**Fig. 2:**
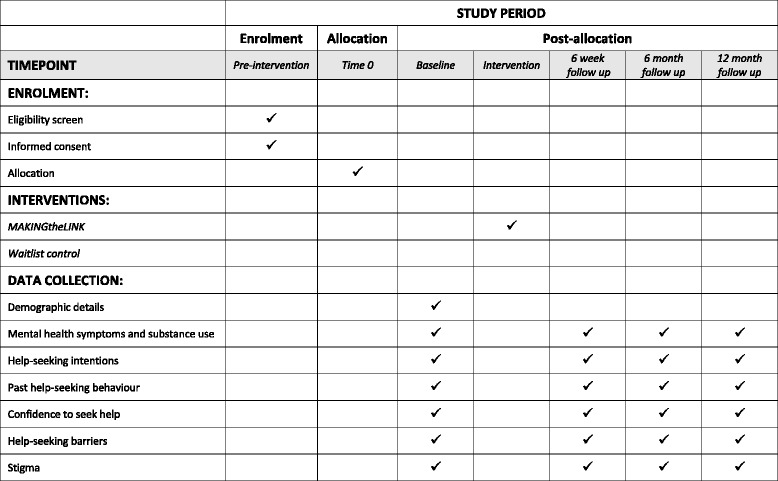
SPIRIT diagram. SPIRIT, Standard Protocol Items: Recommendations for Interventional Trials

All consenting students subsequently undergo baseline testing during class prior to intervention delivery. Intervention schools then receive the programme in their regular class time. Participants will be re-tested the week following the intervention or at an equivalent time for the control schools (6 weeks post baseline). Follow-up assessments will also be completed online during class at 6 and 12 months post baseline. All students will be provided with information on how to seek help for substance use, depression and other problems from a range of resources and services at the completion of the surveys. Figure 2 shows the SPIRIT diagram for the trial procedure.

All information in the survey will be de-identified and confidential, and the records will be kept securely for 5 years at the research centre (Turning Point) after which time they will be destroyed. However, if students report high scores on a screen for depression the researchers will notify the student welfare coordinator (SWC) who will contact the child to check that they are safe and refer for assistance as appropriate. Any student who becomes upset while completing the surveys can stop at any time. The researcher will be available to provide debriefing, and the student will be encouraged to talk to their parents or one of their teachers. They will also be provided with contact details for services, if necessary.

### Measures

In addition to demographic information (age, gender, postcode, language spoken at home, living arrangements, parental occupation, country of birth), the following measures will be administered.

#### Mental health symptoms

Levels of stress, anxiety and depression symptoms will be measured by the 21-item version of the Depression Anxiety Stress Scales (DASS-21) [23]. The DASS-21 consists of 21 statements that measure symptoms of depression, anxiety and stress that are experienced in the past week (seven statements per scale). Each statement is rated on a 4-point scale (0 = not at all, 3 = very much, or most of the time). Scores for each item are summed to indicate participants’ levels of depression, anxiety or stress. The DASS-21 has good discriminant and concurrent validity, sound convergent reliability with other measures of depression and anxiety (e.g. [23, 24]), and has been used successfully in previous studies with adolescent samples [25].

#### Alcohol use

Alcohol use will be measured by adapting questions from the Australian Secondary School Students Alcohol and Drug (ASSAD) Survey [26]. This will allow for comparison between use in the current sample and a large-scale representative group of Australian secondary school students. Participants self-report whether they have ever drunk alcohol or drank in the past year, frequency of drinking and drinks consumed in the past 7 days. Alcohol-related harms will be assessed using questions from two large adolescent longitudinal studies, the Adolescent Temperament Project [27] and the International Youth Development Study [28]. Over 10 questions, participants are asked to indicate on a 3-point Likert scale (0 = never, 1 = once or twice, 2 = more often) the number of times in the last 6 months that their alcohol use had caused them specific problems (e.g. get so drunk they were sick or passed out; have trouble at home, work or school the next day; get injured or have an accident; be unable to remember what happened the night before).

#### Help-seeking

*Intention to seek help* will be measured with the General Help Seeking Questionnaire (GSHQ-V) [29]. This 15-item questionnaire requires participants to indicate how likely they would be to seek help for alcohol or depression from a number of sources (e.g. boyfriend/girlfriend, friend, parent, teacher, GP) rated on a 5-point (1 = very unlikely to 5 = very likely) scale. The problem-type and the help-sources of the GHSQ-V will be modified to ensure that the measure is relevant to the particular context and research questions of the current study in line with recommendations by Wilson and colleagues [30].

*Prior help-seeking behaviour* will be assessed using supplementary questions from the GHSQ-V. Young people will be asked if they have ever sought help from a professional helper (e.g. school counsellor, counsellor, GP, psychologist, psychiatrist, nurse, alcohol and drug worker) and to indicate whether it was for an alcohol, drug, mental health or ‘other’ problem and how helpful this was (rated 1 = very unhelpful to 5 = very helpful). Actual help-seeking in the past 6 months will be assessed with a simplified version of the Actual Help Seeking Questionnaire (AHSQ) [31], and adapted to include substance use and mental health (four items): (1) Have you sought help or advice for an alcohol-related problem in the past 6 months? (2) If yes, who from? (3) Have you sought help or advice for depression or another emotional problem in the past 6 months? (4) If yes, who from? Facilitation of help-seeking for a friend will also be assessed at each time point using a modified version of these tools.

*Confidence to seek help* will be measured using a 5-point Likert scale, where 1 = not confident at all to 5 = very confident: How confident are you to seek help if you had an alcohol or drug problem*?* How confident are you to seek help if you had depression or another emotional problem?

*Confidence to seek help for a peer* will be measured using a 5-point Likert scale, where 1 = not confident at all to 5 = very confident: How confident are you to assist a friend to seek help if they had an alcohol or drug problem? How confident are you to assist a friend seek help if they had depression or another emotional problem?

#### Psychological barriers

*Beliefs about seeking professional help* will be measured using a brief version of the Barriers to Adolescents Seeking Help questionnaire (BASH-B) [32], which comprises 11 barriers to seeking help rated on a 6-point scale from ‘strongly agree’ to ‘strongly disagree’.

*Stigma* will be measured using the Depression Stigma Scale and the Social Distance Scale reported in Yap and colleagues [33]. Mental illness vignettes relevant to alcohol misuse and depression will also be included followed by the questions: ‘How much do you agree with the statement “Sarah needs help”’, ‘How much do you agree with the statement “Sarah would benefit from professional help”’, ‘How confident would you feel to help Sarah see a professional?’ and ‘If you were behaving like Sarah, what do you think could be wrong with you?’ The first four questions are scored on a Likert scale and the fifth is a free-text response.

### Blinding

Schools will be unaware of each school’s allocation until after they have provided consent. Participating individuals and their parents will be unaware of whether they have been allocated to the intervention or control group until after the first data collection point.

### Statistical analysis

**Fig. 3:**
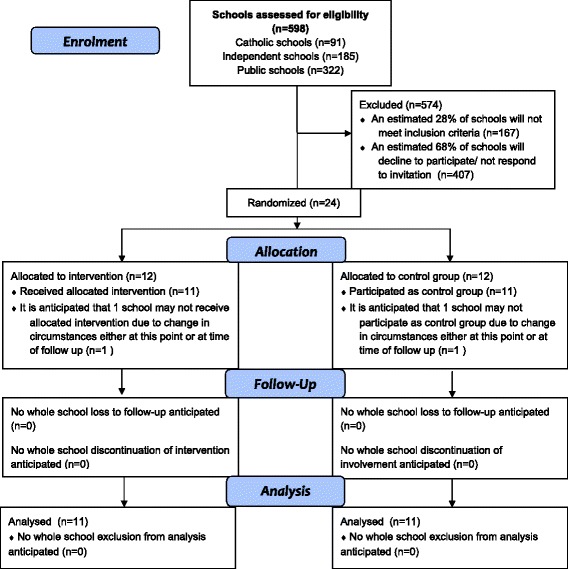
Consolidated Standards of Reporting Trials (CONSORT) flow diagram of progress through the phases of the MAKINGtheLINK trial

The research staff will co-ordinate all appropriate data management and cleaning prior to analysis. Data on screening, refusals and dropout will be coded and reported as per Consolidated Standards of Reporting Trials (CONSORT) guidelines for participant flow through the trial (see Fig. 3). A description of the baseline characteristics of schools and of individuals who participate in the two intervention arms will be compiled using descriptive statistics such as mean and standard deviation, median and interquartile range, and percentages.

In order to evaluate the main aim of the study, which is to examine the association between participation in the help-seeking programme (control versus intervention arm) and actual help-seeking behaviour at 12 months post intervention, logistic regression analyses will be undertaken testing for group differences between help-seeking behaviour as measured by the AHSQ. In a secondary analysis, gender, depression and anxiety scores, reported alcohol problems, and any characteristics of schools or participants that are imbalanced to a large extent at baseline, will be included in an extended logistic regression model for help-seeking behaviour in addition to the control/intervention arm indicator variable.

For secondary aims, the outcomes are measured by continuous variables, and as such linear regression analysis will be used to compare the two study arms with the primary analysis comprising models with treatment indicator and the baseline value of the outcome score, i.e. analysis of covariance. Secondary analyses will adjust for imbalanced characteristics in the same way as for the primary analysis. All analyses will utilise robust standard errors to adjust for clustering by school.

Each analysis described above will be conducted using participants with all necessary data for that analysis, according to the intention-to-treat principle. Characteristics of participants who were lost to follow-up and who completed follow-up will be compared. Analyses will be repeated using all participants on the basis of multiple imputation involving responses at all four time points to impute for missing values.

## Discussion

In recent years, government policies and strategies have shifted from a focus on treatment to prevention and early intervention, particularly for alcohol and depression. However, despite considerable investment in early intervention services for young people in Australia, more than 75 % of 16–24 year-olds do not access professional help for a mental health or substance use disorder [2]. Instead, young people keep their problems to themselves or turn to their peers, with multiple barriers to help-seeking consistently identified among adolescents [7]. The aim of the current trial is to establish the efficacy of a universal school-based intervention that focusses on improving help-seeking and peer support for students experiencing mental health and/or substance use problems, by examining the impact of the intervention on subsequent help-seeking attitudes, confidence, intentions and behaviour.

A particular strength of the study design is its focus on actual help-seeking behaviours, as opposed to help-seeking intentions alone. Measuring behaviour is critical in establishing the efficacy of help-seeking interventions, as while intentions to seek help are a good indicator of subsequent behaviour [29, 30], they do not accurately demonstrate how adolescents actually respond to real-life events. As previous research indicates that adolescents are more likely to rely on family and friends when experiencing mental health problems than to seek professional help, we aim to examine help-seeking behaviour from both informal and formal sources. However, it is also important to note that some participants will not experience any problems over the duration of the trial, but may need to seek help at a later time point. Understanding the impact of the intervention on psychological barriers and confidence is necessary in this regard, as improving attitudes towards help-seeking is likely to facilitate future help-seeking behaviour.

A cluster randomised approach was adopted as this has the advantage of controlling for potential contamination of information between individuals in the same setting (i.e. between control and intervention students in the same school). Our previous experience with MAKINGtheLINK is that the lessons learnt in the classroom are often embraced by the school community, and as such, individual randomisation within a school setting would have created additional confounds. Including measures of school disadvantage, as well as other demographic and clinical data, provides an opportunity to explore predictors of help-seeking outcomes within particular school environments, as well as how the intervention is influenced by current mental health symptoms and past help-seeking behaviour.

A number of changes were made to the programme that built on the findings from the two pilot studies. These studies demonstrated that the programme was both highly feasible and acceptable within school settings [15], and led to an initial reduction in help-seeking barriers and increase in intentions to seek help from formal sources [16]. However, these effects were not consistently maintained at follow-up 6 weeks later, as participants’ help-seeking intentions had returned to baseline levels at this time point. In response to these findings, the programme was expanded to include the addition of a booster session 1 month after the initial intervention, given evidence that this can increase retention of knowledge and improve outcomes. The booster session provided students with a second opportunity to practice help-seeking skills using a different video scenario of a young person struggling with personal issues, as well as a poster-making activity that provided students with the opportunity to share their knowledge with other year groups. Materials from the two previous pilots were further refined, with additional mental health examples and case studies added to the programme, as well as a discussion of the mental health continuum, which increased the length of the overall intervention. Teacher training was offered to staff across the school, and a newsletter was provided to staff to ensure that they were aware of the agreed referral process within the school and were able to provide a helpful response if approached by students.

Mid-adolescence is an important period in regard to help-seeking, as mental health and substance use issues become more prevalent and young people increasingly turn to their peers for support. The MAKINGtheLINK programme was designed to address a number of critical gaps in existing early intervention and health promotion activities by teaching school students how to overcome barriers associated with seeking help, as well as how to effectively support their peers. This trial will establish the effectiveness of the MAKINGtheLINK programme and, if found to be successful, support its adoption with a national school framework.

## Trial status

Recruitment commenced in August 2013. The trial is currently underway. The estimated completion date is mid-late 2016.

## Abbreviations

AHSQ, Actual Help Seeking Questionnaire; ANZCTR, Australia and New Zealand Clinical Trials Register; ASSAD, Australian Secondary School Students Alcohol and Drug Survey; BASH-B, Barriers to Adolescents Seeking Help scale; CONSORT, Consolidated Standards of Reporting Trials; DASS, Depression Anxiety Stress Scales; GHSQ-V, General Help Seeking Questionnaire; ICC, intra-class correlation; ICSEA, Index of Community Socio-Educational Advantage; IMB, Information-Motivation-Behavioural Skills Model; RCT, randomised controlled trial; SPIRIT, Standard Protocol Items: Recommendations for Interventional Trials; SWC, student welfare coordinator; TpB, Theory of Planned Behaviour

## Additional file

Additional file 1: Populated SPIRIT checklist. (DOCX 1353 kb)
