# Supplementary figures and images for: A school-based health promotion programme to increase help-seeking for substance use and mental health problems: study protocol for a randomised controlled trial
Source: Trials. 2016 Aug 8;17:393. doi: 10.1186/s13063-016-1510-2 (PMC4976510; doi:10.1186/s13063-016-1510-2)

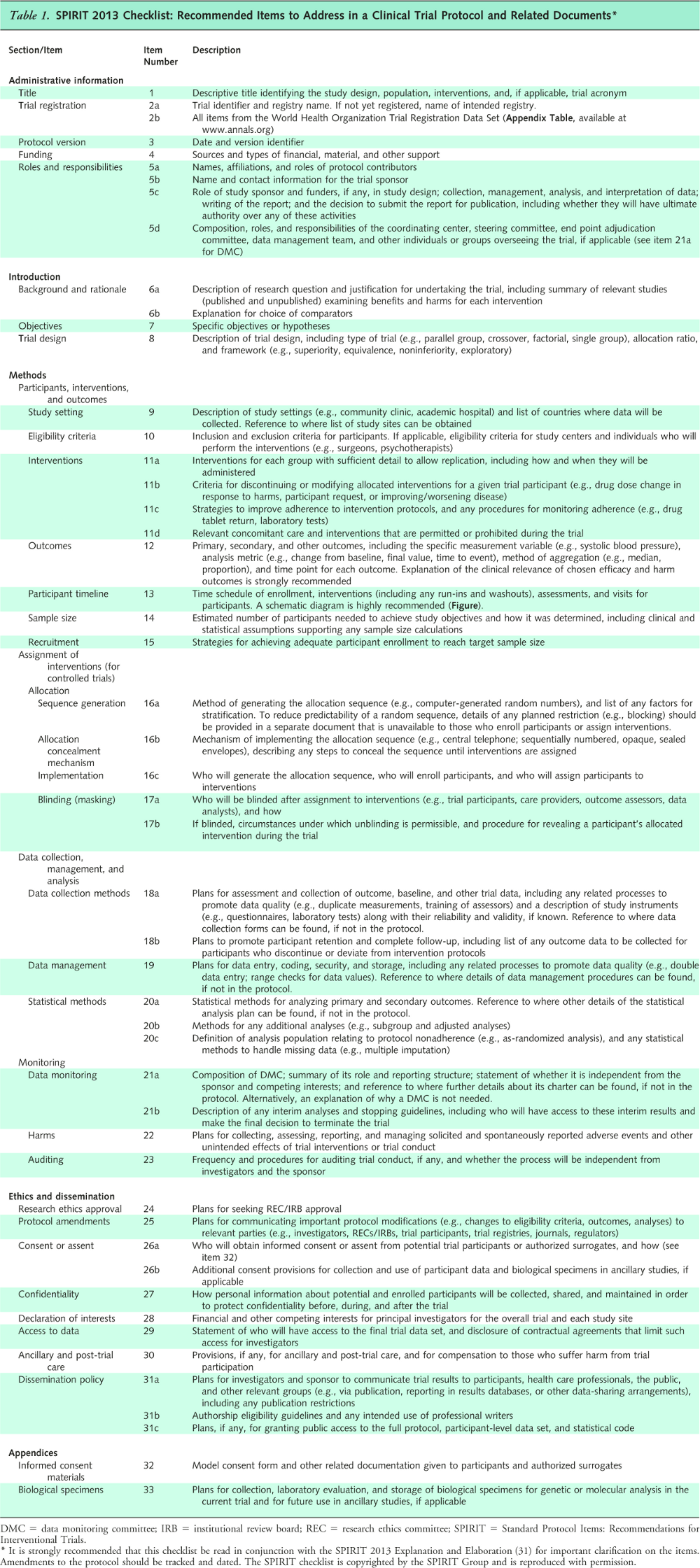

Supplement: Additional file 1: — Populated SPIRIT checklist. (DOCX 1353 kb) [file 13063_2016_1510_MOESM1_ESM.docx]
